# Potential Therapeutic Benefit of C1-Esterase Inhibitor in Neuromyelitis Optica Evaluated *In Vitro* and in an Experimental Rat Model

**DOI:** 10.1371/journal.pone.0106824

**Published:** 2014-09-05

**Authors:** Lukmanee Tradtrantip, Nithi Asavapanumas, Puay-Wah Phuan, A. S. Verkman

**Affiliations:** Departments of Medicine and Physiology, University of California San Francisco, San Francisco, California, United States of America; Kyushu University, Japan

## Abstract

Neuromyelitis optica (NMO) is an autoimmune demyelinating disease of the central nervous system in which binding of anti-aquaporin-4 (AQP4) autoantibodies (NMO-IgG) to astrocytes causes complement-dependent cytotoxicity (CDC) and inflammation resulting in oligodendrocyte and neuronal injury. There is compelling evidence for a central role of complement in NMO pathogenesis. Here, we evaluated the potential of C1-esterase inhibitor (C1-inh) for complement-targeted therapy of NMO. C1-inh is an anti-inflammatory plasma protein with serine protease inhibition activity that has a broad range of biological activities on the contact (kallikrein), coagulation, fibrinolytic and complement systems. C1-inh is approved for therapy of hereditary angioedema (HAE) and has been studied in a small safety trial in acute NMO relapses (NCT 01759602). In vitro assays of NMO-IgG-dependent CDC showed C1-inh inhibition of human and rat complement, but with predicted minimal complement inhibition activity at a dose of 2000 units in humans. Inhibition of complement by C1-inh was potentiated by ∼10-fold by polysulfated macromolecules including heparin and dextran sulfate. In rats, intravenous C1-inh at a dose 30-fold greater than that approved to treat HAE inhibited serum complement activity by <5%, even when supplemented with heparin. Also, high-dose C1-inh did not reduce pathology in a rat model of NMO produced by intracerebral injection of NMO-IgG. Therefore, although C1r and C1s are targets of C1-inh, our in vitro data with human serum and in vivo data in rats suggest that the complement inhibition activity of C1-inh in serum is too low to confer clinical benefit in NMO.

## Introduction

Neuromyelitis optica (NMO) is autoimmune disease of the central nervous system in which inflammatory demyelinating lesions cause optic neuritis and transverse myelitis [1], [2]. Most NMO patients are seropositive for immunoglobulin G autoantibodies (NMO-IgG) against aquaporin-4 [3], [4], a water channel expressed on the plasma membrane of astrocytes [5]. NMO pathogenesis in patients seropositive for NMO-IgG involves NMO-IgG binding to astrocyte AQP4, causing cytotoxicity with secondary inflammation, oligodendrocyte injury and demyelination [6], [7]. Currently used NMO therapies include immunosuppressives, B-cell depletion and plasma exchange [8]–[10].

There is strong evidence for a central role of complement in NMO pathogenesis and hence for the utility of complement-targeted therapeutics. Inflammatory lesions in human NMO show prominent vasculocentric deposition of activated complement [11]–[13]. In vitro, addition of NMO-IgG and complement to AQP4-expressing cells, including astrocytes, produces complement-dependent cytotoxicity (CDC) [14]–[17]. Characteristic NMO pathology with demyelination is produced in ex vivo spinal cord slice cultures exposed to NMO-IgG and human complement [18], and in mice in vivo following intracerebral injection of NMO-IgG and human complement [19]. In rats, which have an active complement system similar to that in humans, administration of NMO-IgG alone causes complement-dependent NMO pathology, as pathology is not seen when complement is inactivated by cobra venom toxin or when NMO-IgG is mutated to block its complement effector function [20]. An open-label clinical trial of eculizumab, a monoclonal antibody inhibitor targeting complement protein C5, showed reduced recurrence rate in NMO patients with severe disease [21].

**Figure 1:**
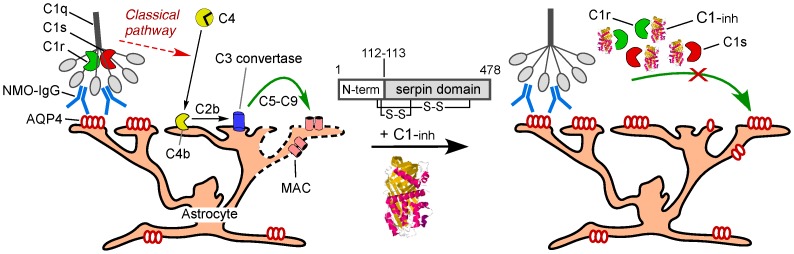
Action of C1-inh on NMO-IgG-dependent cytotoxicity involve the classical complement pathway. NMO-IgG binds to cell surface AQP4 on astrocytes, which activates the classical complement pathway by C1q binding to the Fc region of NMO-IgG leading to formation of the membrane attack complex (MAC) (left). C1-inh binds to and inactivates C1r and C1s, preventing C1q action on C4 and downstream complement activation.

Though further clinical evaluation of eculizumab in NMO is awaited, there is interest in the development of alternative complement-targeted therapeutics in NMO as eculizumab is very costly and associated with significant infectious complications including meningococcal meningitis [22]. Our lab recently introduced complement-targeted monoclonal therapeutics that target C1q and C1s in the classical complement pathway [23]. Selective inhibition of early steps in the classical complement pathway has potential benefit over inhibition of later steps because the lectin activation pathway, which is involved in bacterial killing, remains intact. There has been interest in the therapeutic potential of C1-esterase inhibitor (C1-inh), an anti-inflammatory plasma protein with serine protease inhibition activity and a wide range of biological activities on the contact (kallikrein), coagulation and fibrinolytic systems, and on the complement pathway (Fig. 1) [24], [25]. Purified C1-inh from human serum is approved for use in hereditary angioedema (HAE) based on its kallikrein inhibition activity, and recently, based on its known complement inhibition activity, a safety trial (NCT 01759602) has been completed for acute NMO relapses [26]. In that trial safety was demonstrated in ten patients administered 2000 units of C1-inh daily for three days. Here, utilizing in vitro and rat model systems, we evaluated the potential utility of C1-inh therapy for NMO.

## Materials and Methods

### Cell culture and antibodies

Chinese hamster ovary (CHO) cells stably expressing M23-AQP4 were used, as described [27]. CHO cells were cultured in F-12 Ham's Nutrient mix medium supplemented with 10% fetal bovine serum, 100 units/ml penicillin, 100 µg/ml streptomycin, and 200 µg/ml geneticin (as selection marker). Cells were grown at 37°C in 5% CO_2_/95% air. Recombinant monoclonal NMO antibody rAb-53 (referred to as NMO-IgG) was generated from clonally expanded plasma blasts from cerebrospinal fluid of NMO patients and purified, as described [17].

### Rats

Lewis rats were purchased from Charles River Lab (Wilmington, MA). Experiments were done using weight-matched rats (250–350 g). Protocols were approved by Institutional Animal Care and Use Committee (IACUC) at the University of California San Francisco (Project Number: AN89748). All surgery was performed under ketamine/xylazine anesthesia, and analgesics were used post-operatively to minimize pain.

### Complement-dependent cytotoxicity (CDC)

M23-AQP4-expressing CHO cells were plated onto 96-well microplates (Costar, Corning Inc., Corning, NY) at 25,000 cells/well and grown for 24 h. Cells were washed with phosphate-buffered saline (PBS) and incubated at room temperature for 60 min with NMO-IgG or purified IgG from NMO patient serum in live cell buffer (PBS containing 6 mM glucose and 1 mM sodium pyruvate) containing pooled normal human complement serum (Innovative Research, Novi, MI) with or without C1-inh (Berinert, CSL Behring, King of Prussia, PA) in a final volume of 50 µl. Some experiments were done with rat serum, prepared from freshly obtained rat blood and then frozen, in place of the human complement serum. In some experiments, a solution containing pooled normal human complement serum was incubated with C1-inh for specified times before addition to the CHO cells. To measure cytotoxicity, cells were washed twice with PBS, and incubated with 50 µl of a 20% Alamar Blue solution (Invitrogen) for 45 min at 27°C. Cytotoxicity was measured from resorufin fluorescence using a TECAN Infinite M1000 plate reader (TECAN Groups Ltd, Mannedorf, Switzerland) (excitation/emission 560/590 nm).

### C1-inh administration in rats and serum collection

Adult rats (250–350 g) were injected by tail vein with C1-inh (600 units/kg) in saline in a total volume of 500 µl. Blood was collected through the tail vein at 0.25, 0.5, 1, 2, 4, 6, and 8 h, left for 30 min at room temperature to allow clotting, and centrifuged for 10 min at 3000 g, 4°C. Serum was collected and bioassayed for CDC by addition of 2% rat serum and 1 µg/ml NMO-IgG to M23-AQP4-expressing CHO cells for 60 min. Cytotoxicity was measured by the Alamar Blue assay. In some experiments heparin (1.5 mg/kg) was injected by tail vein with or without C1-inh (600 units/kg) in saline in a total volume of 500 µl.

### Rat model of NMO

A rat intracerebral injection was used, as described [20]. Briefly, adult rats were anesthetized with intraperitoneal ketamine (75–100 mg/kg) and xylazine (5–10 mg/kg) and mounted in a stereotaxic frame. Following a midline scalp incision, a burr hole of diameter 1 mm was made in the skull 3.5 mm to the right of the bregma. A 30-gauge needle attached to 50-µl gas-tight glass syringe was inserted 5 mm deep to infuse 10 µg NMO-IgG in a total volume of 10 µl (at 2 µl/min). Rats were injected intravenously with 300 units/kg C1-inh in saline (or saline alone) in a total volume of 300 µl just before and 1 day after intracerebral injection of NMO-IgG. After 2 days, rats were anesthetized and perfused through the left cardiac ventricle with 100 ml PBS and then 25 ml of PBS containing 4% paraformaldehyde. Five micrometer-thick paraffin sections were immunostained for 1 h with antibodies against rat AQP4 (1∶200, Santa Cruz Biotechnology), GFAP (1∶100, Millipore) and myelin basic protein (MBP) (1∶200, Santa Cruz Biotechnology), followed by the appropriate fluorescent secondary antibody (1∶200, Invitrogen). Tissue sections were examined with a Leica (Wetzlar, Germany) DM 4000 B microscope at 2.5× magnification. AQP4, GFAP and MBP immunonegative areas were defined by hand and quantified using ImageJ. Data are presented as area (mm^2^) of immunonegative area.

### Statistical analysis

Data are presented as mean ± S.E. Comparisons between two groups were performed using Student's t-test.

## Results

### C1-inh inhibits NMO-IgG-dependent CDC

**Figure 2:**
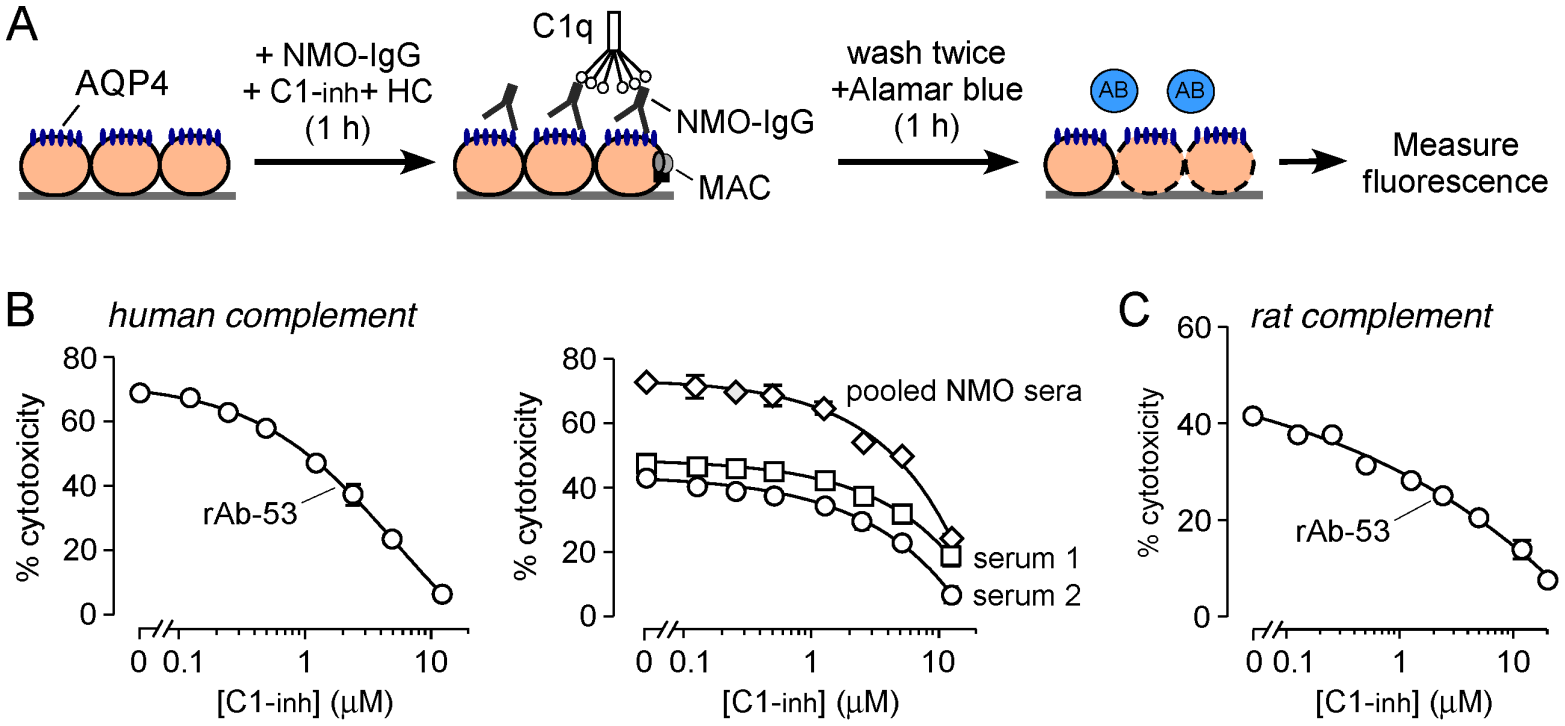
C1-inh inhibits complement-dependent cytotoxicity produced by NMO-IgG in AQP4-expressing cell cultures. A. CDC assay showing 1 h incubation of NMO-IgG, human complement (HC) and C1-INH to CHO cells expressing human M23-AQP4, followed by assay of cytotoxicity with AlamarBlue. B. Percentage cytotoxicity as a function of C1-inh concentration in cells incubated with human complement (2%) and recombinant NMO-IgG rAb-53 (1 µg/ml) (left) or individual or pooled human NMO sera (100 µg/ml) (right). C. Study as in B in cells incubated with rAb-53 (0.5 µg/ml) and rat serum (2%). Data are mean ± S.E. for 4 measurements per condition.

To study C1-inh inhibition of NMO-IgG-dependent CDC, cytotoxicity was assayed in CHO cells expressing AQP4 after incubation with NMO-IgG and human complement, as diagrammed in Fig. 2A. Fig. 2B shows concentration-dependent C1-inh inhibition of cytotoxicity in cells incubated with 2% human complement and a recombinant monoclonal NMO-IgG (rAb-53) (left) and sera from two NMO patients and pooled NMO patient sera (right). C1-inh inhibited cytotoxicity in a concentration-dependent manner, with 50% protection at ∼ 2.5–5 µM C1-inh at 2% human complement. Fig. 2C shows C1-inh protection from cytotoxicity in cells incubated with rAb-53 and 2% rat serum.

**Figure 3:**
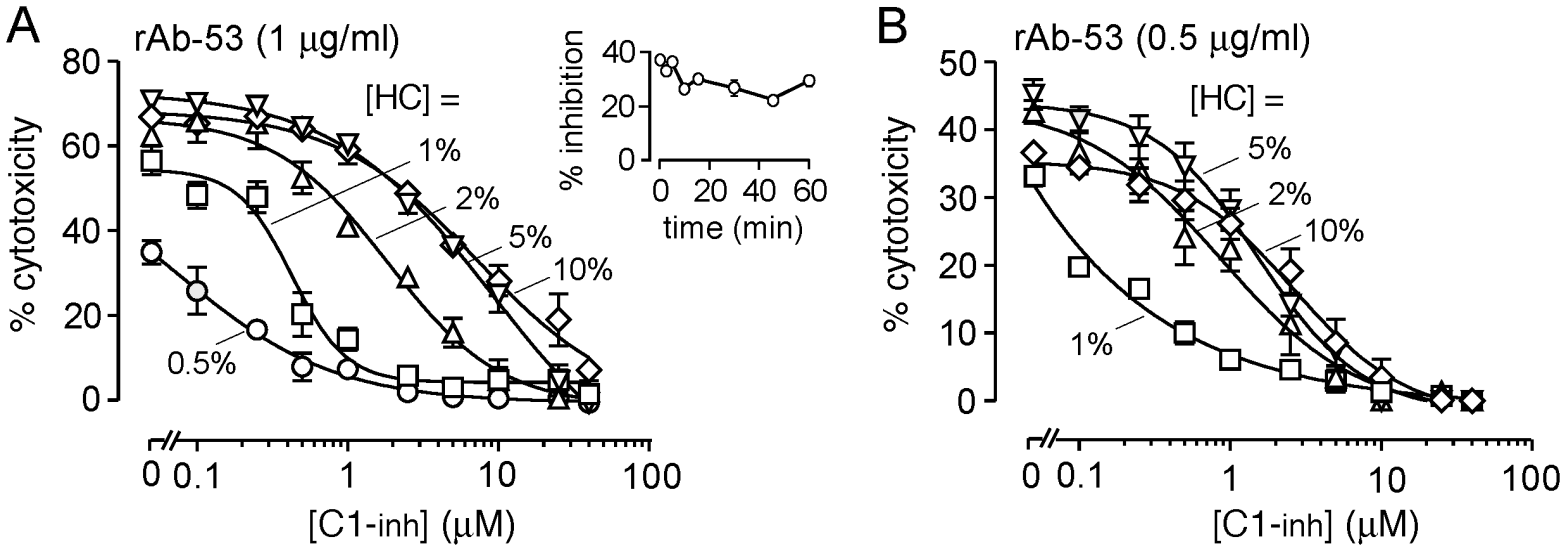
C1-inh inhibition of NMO-IgG-dependent cytotoxicity depends on complement and NMO-IgG concentration. A. Percentage cytotoxicity as a function of C1-inh concentration in cells incubated with 1 µg/ml rAb-53 and indicated concentration of human complement. Data are mean ± S.E. for 4 measurements per condition. Inset shows percentage inhibition of cytotoxicity as a function of time after pre-incubation of 2 µM C1-inh and 2% human complement prior to addition AQP4-expressing CHO-cell that were pre-incubated with 1 µg/ml rAb-53. B. Study as in A but with 0.5 µg/ml rAb-53.

Because C1-inh action involves blocking the classical complement pathway by irreversible inactivation of enzymes C1r and C1s in the C1 complex [28], [29], we investigated the efficacy of C1-inh inhibition on NMO-IgG-dependent CDC as a function of complement concentration. Fig. 3A, B shows concentration-dependent C1-inh inhibition with increasing complement, shown for two different NMO antibody concentrations. At an antibody concentration of 1 µg/ml, 7 µM C1-inh produced ∼50% inhibition of CDC at 10% human complement. Though it is not possible to test undiluted human serum or higher concentrations of human complement in this assay, a much higher concentration of C1-inh, perhaps as high as 70 µM (estimated by multiplying 7 µM IC_50_ at 10% human complement by ten to extrapolate to undiluted serum), may be required to produce 50% inhibition of CDC with undiluted (100%) human serum. Given the equivalence of 1 unit/mL C1-inh to 2.5 µM, a 2000 unit dose of C1-inh administrated to a human with 5000 mL blood volume could produce a maximum increase of serum C1-inh of 0.4 units/mL (or 1 µM), much less than 70 µM. The inset to Fig. 3A shows that the inhibition of CDC by C1-inh is rapid.

### Polysulfated macromolecules increase C1-inh inhibition potency

**Figure 4:**
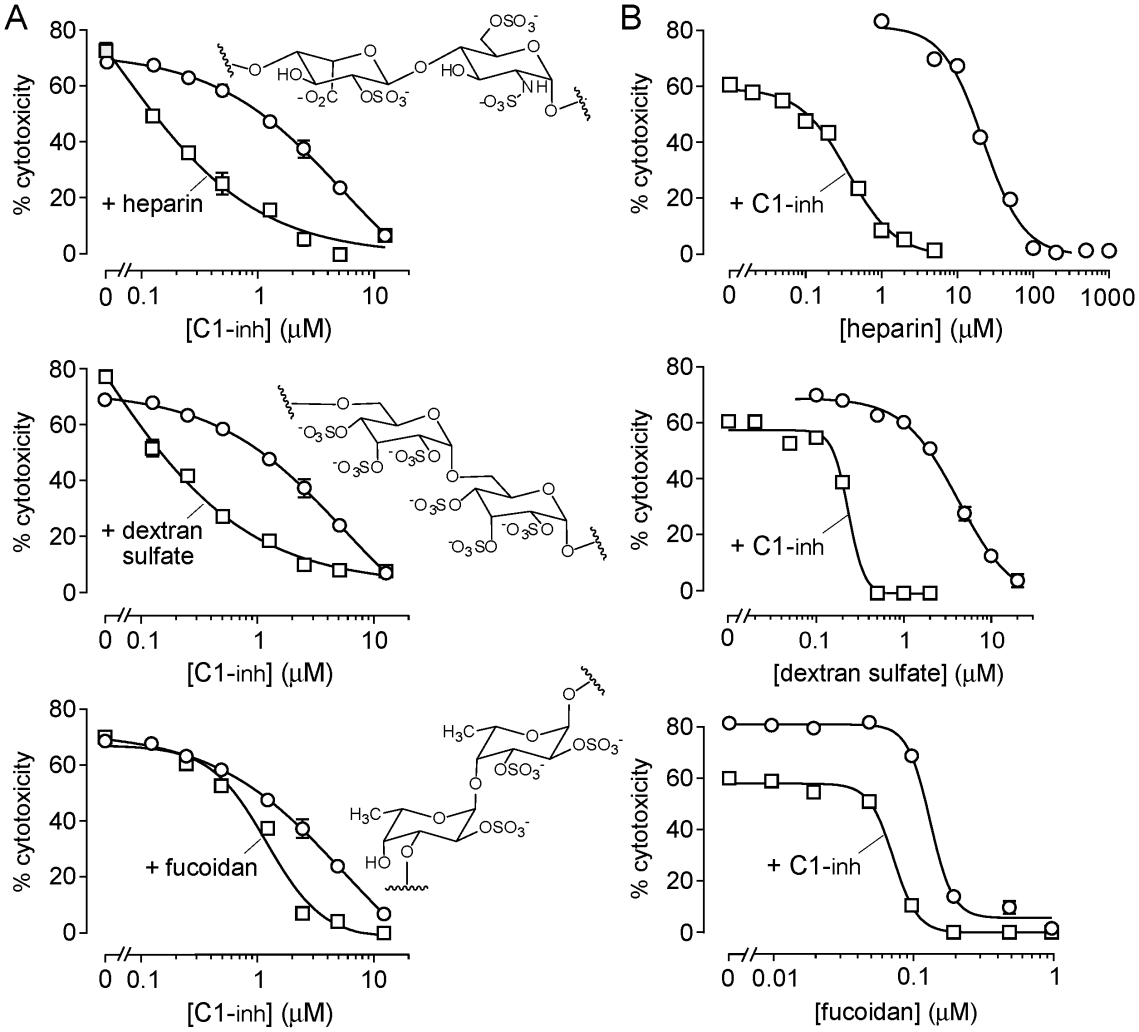
Polysulfated macromolecules potentiate C1-inh inhibition of NMO-IgG-dependent CDC. A. Percentage cytotoxicity as a function of C1-inh concentration in cells incubated with human complement (2%) and rAb-53 (1 µg/ml) in the absence or presence of 2 µM heparin, 0.5 µM dextran sulfate or 0.02 µM fucoidan (mean ± S.E., n = 4). Insets show chemical structures. B. Percentage cytotoxicity as a function of concentration of polysulfated macromolecules in cells incubated with human complement (2%) and rAb-53 (1 µg/ml) in the absence or presence of 1 µM C1-inh.

Based on the in vitro data showing weak C1-inh complement inhibition, we attempted to augment and potentiate the complement inhibition action of C1-inh using macromolecules, such as heparin, that inhibit complement directly [30], possibly by multivalent interaction with C1q that impair its binding to the antibody Fc region [31], [32]. Polysulfated macromolecules are also reported to potentiate the action of C1-inh [33], possibly by direct binding to the contact area of the protease – C1-inh complex [34]. We studied the complement inhibition action and potentiation of C1-inh activity in vitro by three polysulfated macromolecules – heparin, dextran sulfate, and the nutraceutical fucoidan. Fig. 4A shows that each of the three polysulfated macromolecules potentiated C1-inh inhibition of NMO-IgG-dependent CDC, reducing the C1-inh concentration needed to produce inhibition, by nearly 10-fold for heparin and dextran sulfate. Fig. 4B shows that each of the polysulfated macromolecules alone inhibited CDC, with substantial potentiation seen at a low concentration of C1-inh. These results suggest the potential synergistic use of C1-inh and heparin to enhance complement inhibition activity in vivo, as investigated further below.

### C1-inh administration to rats produces little inhibition of serum complement activity

**Figure 5:**
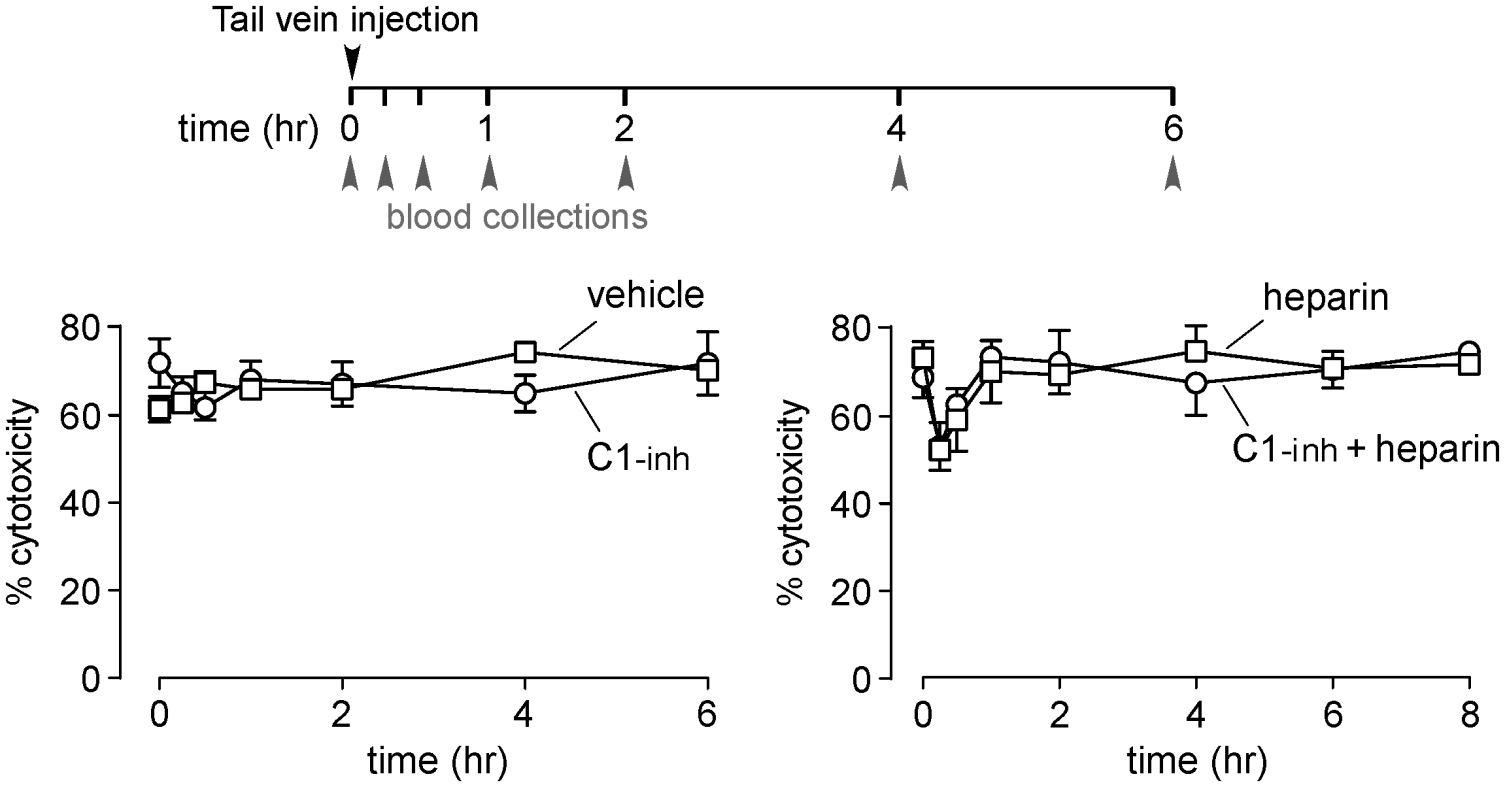
Intravenous administration of high-dose C1-inh in rats does not inhibit complement activity. A. C1-inh was administered to rats by tail vein injection, and serum samples obtained at specified times were bioassayed for NMO-IgG-dependent CDC by incubation of AQP4-expressing CHO-cells with 2% rat serum with 1 µg/ml rAb-53 on percentage cytotoxicity produced by rat serum at different times after administration of 600 units/kg C1-inh (or vehicle control) (left), and 1.5 mg/kg heparin, without or with 600 units/kg C1-inh (right) (mean ± S.E., 4 rats per condition).

To investigate the complement inhibition activity of C1-inh in rats, C1-inh was administered intravenously, and serum obtained at different times was assayed for complement activity. Initial studies done using doses used to treat HAE in humans (20 units/kg) did not show inhibition of complement activity in rat serum. We therefore increased the C1-inh dose by 30-fold (to 600 units/kg). However, no significant inhibition of rat serum complement activity was found at any time after C1-inh administration at this high dose (Fig. 5). The presence of multiple C1-inh substrates in serum could contribute the poor inhibition potency of C1-inh in vivo (see Discussion).

Given the results above showing that heparin and related polysulfated macromolecules can potentiate the inhibitory effect of C1-inh on CDC in vitro, we administered rats high-dose C1-inh and heparin together. However, as shown in Fig. 5, there was only minimal and transient reduction in complement activity in rat serum in both heparin-treated groups, with no potentiation produced by C1-inh.

### C1-inh administration to rats does not reduce NMO pathology

**Figure 6:**
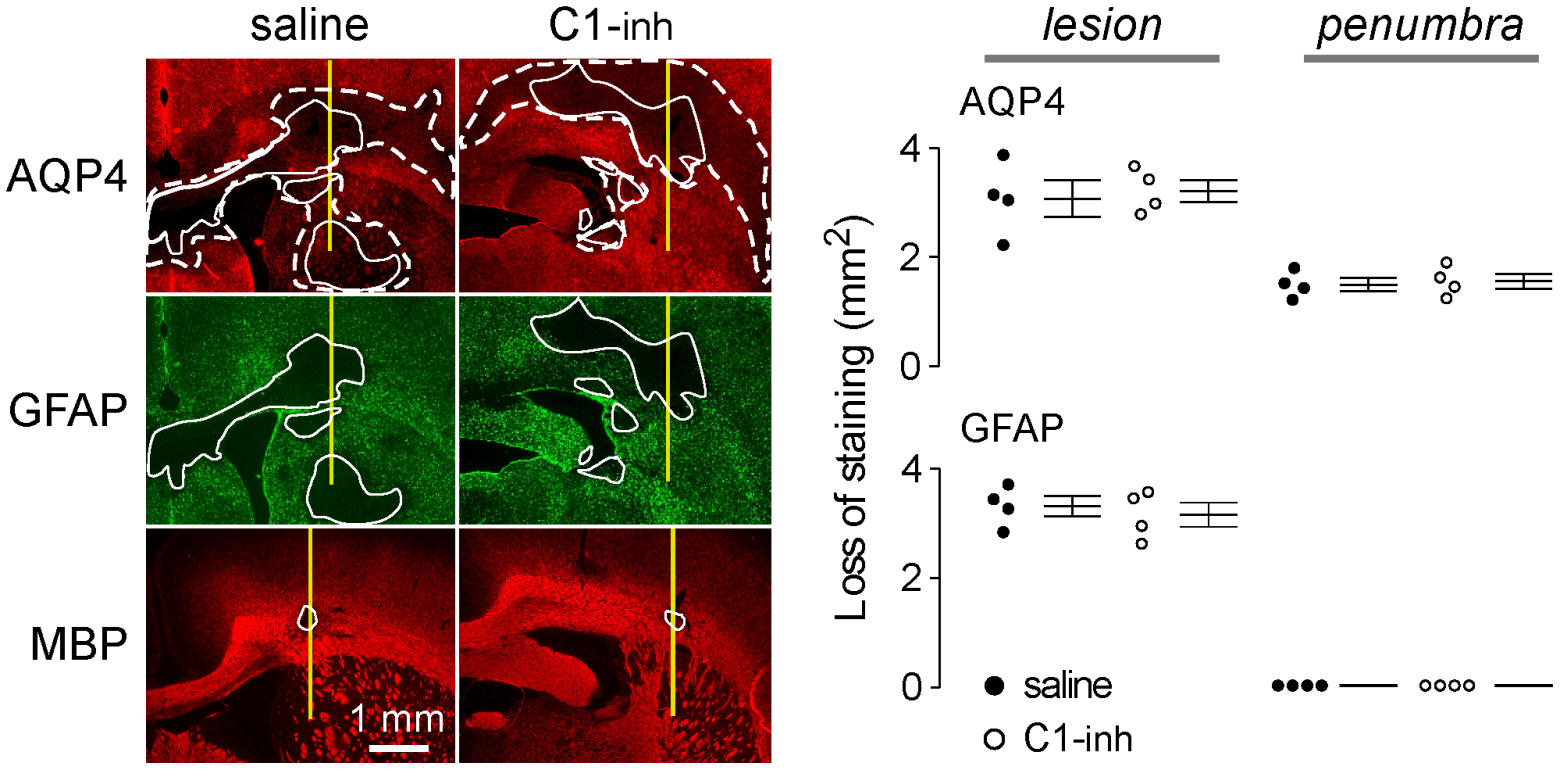
High-dose C1-inh does not reduce brain pathology in a rat model of NMO produced by intracerebral injection NMO-IgG. AQP4, GFAP and MBP immunofluorescence at 2 days after injection of 10 µg rAb-53. C1-inh (300 units/kg) (or saline control) was administered intravenously just before and 1 day after intracerebral injection of NMO-IgG. The needle tract is shown in yellow line, the white line demarcates lesion areas with loss of AQP4 and GFAP immunofluorescence, and the white dashed line demarcates the penumbra areas with reduced AQP4 but normal GFAP immunofluorescence (left). Summary of lesion areas showing data for individual rats (mean ± S.E., n = 4). Differences not significant.

We used an established rat model of NMO to test the in vivo efficacy of C1-inh. Though the lack of complement inhibition in rat serum would predict no significant clinical benefit in a rat model of NMO, the complement data do not rule out other actions of C1-inh that might be beneficial in NMO. In our model NMO pathology in brain is produced by a single intracerebral injection of NMO-IgG, which results in characteristic pathological features of NMO including astrocyte damage (loss of AQP4 and GFAP immunoreactivity), inflammation and demyelination by 5 days [20]. Pathology in this model was fully prevented by complement inhibition with cobra venom toxin [20], indicating that rat complement activity is required to produce pathology. Also, NMO pathology in this rat model was partially (∼50%) reduced by intravenous immunoglobulin G [35], indicating its sensitivity to disease-modifying interventions. Here, C1-inh (or saline as control) was administered intravenously at high dose (300 units/kg daily for 2 days after intracerebral injection with NMO-IgG). Fig. 6 (left) shows that C1-inh did not protect against NMO pathology produced by NMO-IgG injection at day 2, as seen by large areas with loss of AQP4 and GFAP (white lines) or AQP4 alone (‘penumbra region’, dashed white lines). Fig. 6 (right) summarizes areas of loss of AQP4 and GFAP immunofluorescence.

## Discussion

The complement system is an attractive target for therapy of NMO, as complement-dependent astrocyte cytotoxicity is an early initiating event in the neuroinflammation and demyelination that occurs in NMO. Selective inhibition of early steps in the classical complement pathway is of particular interest because NMO pathogenesis involves NMO-IgG complement effector function in which the antibody Fc region binds C1q to initiate complement activation. We found previously that CDC requires physical clustering of NMO-IgG on astrocytes [27], which is facilitated by the supramolecular assembly of AQP4 molecules in orthogonal arrays of particles [36]. Currently used therapies in NMO, immunosuppressives, B-cell depletion and plasma exchange, do not target the underlying pathogenic events in NMO. Alternative NMO therapeutics under evaluation or development include monocloncal antibodies targeting IL-6 receptors on plasma cells (toculizumab, [37]) and NMO-IgG binding to AQP4 (aquaporumab, [38]), and small molecule inhibitors of neutrophils [39] and eosinophils [40]. We previously found that a novel, C1q-targeted monoclonal antibody blocked CDC in vitro and prevented the development of pathology in an in vivo mouse model of NMO [23]. Evaluation of C1-inh in NMO is motivated by its complement inhibition action on the serine protease activities of C1r and C1s in the classical complement pathway [25], [28], [29]; however, C1-inh inhibition of the complement lectin pathway has also been reported [41] and the serine protease inhibition action of C1-inh affects multiple other biological activities, including the coagulation system, the fibrinolytic system, T-lymphocyte activation and the kallikrein contact activation system [25], [26]. The therapeutic efficacy of C1-inh in HAE involves its action on the kallikrein system [42], [43].

We found here that C1-inh produced modest inhibition of NMO-IgG-dependent CDC in vitro, and that intravenous administration of large amounts of C1-inh in rats did not inhibit rat complement activity nor did it reduce brain pathology in a rat model of NMO. The studies here were done using rats rather than mice because of the weak activity of the mouse classical complement pathway [44]. C1-inh concentration in human serum is ∼2–3 µM, and can increase by 2–2.5 fold with inflammation [4]. HAE patients have a broad range of C1-inh concentrations of 5–38% of normal levels [45]. After treatment with 25 units/kg C1-inh, C1-inh concentration increases to 60–70% of normal levels shortly and is maintained at 40% of normal levels over 24 h [46]. We found here that intravenous administration to rats of substantially greater amounts of C1-inh, 600 units/kg, did not inhibit serum complement activity or reduce NMO pathology produced by intracerebral NMO-IgG administration. These data suggest that C1-inh is unlikely to be of therapeutic benefit in NMO based on its complement inhibition activity.

The recently completed C1-inh safety trial (NCT 01759620) involved intravenous administration of CINRYZ, 2000 units per day for 3 days, in NMO patients undergoing an acute exacerbation [26]. For a 70 kg person this corresponds to 28 units/kg per dose, similar to that used to treat HAE. As discussed above, this dose of C1-inh is predicted to produce no significant inhibition of complement activity. In a single case report, a substantially higher dose of C1-inh (6000 units, followed by a total of 7000 units over 2 days) was administered to a 64-year old female with autoimmune hemolytic anemia associated with non-Hodgkin lymphoma [47]. An apparent clinical response was reported, with improved transfusion efficiency and reduced erythrocyte C3 deposition. Potentially such very larges doses of C1-inh could have therapeutic benefit in NMO, though the in vitro and rat data here provide evidence against significant complement inhibition by C1-inh even with such large doses. Human studies are needed to quantify the inhibition of NMO-IgG-dependent cytotoxicity of serum from humans administered high doses of C1-inh.

Given the weak complement inhibition activity of C1-inh, as a possible strategy to potentiate its activity, we investigated the efficacy of polysulfated macromolecules, including heparin, dextran sulfate, and fucoidan. Assays of NMO-IgG-dependent CDC in vitro showed that each of the three polysulfated macromolecules enhanced the inhibitory effect of C1-inh, with ∼10-fold potentiation produced by heparin and dextran sulfate. However, administration of C1-inh and heparin to rats showed no C1-inh -dependent serum complement inhibition.

Though modest C1-inh inhibition of human and rat complement were found in in vitro assays, no significant inhibition of serum complement activity was found in rats in vivo, even after high-dose intravenous C1-inh administration, which translated to lack of efficacy in a rat model of NMO. There are several possible reasons for the lack of C1-inh efficacy on vivo. Whole blood contains many substrates of C1-inh, in addition to C1r and C1s, that are present relatively large amounts, including mannan-binding lectin-associated proteins (MASP-1 and MASP-2), kallikrein, FXIIa and plasmin, which compete for inactivation by C1-inh. C1-inh is a member of a suicidal family of inhibitors, in which a covalent complex is irreversibly formed between C1-inh and its protease target [28], [29]. Though C1-inh can inhibit C1-auto-inactivation in the fluid-phase [48], it is a poor inhibitor of C1-activated immune complexes on membranes [49], [50], as is relevant in NMO. One group reported that the concentration of C1-inh for inhibition of C1 binding to the antibody-sensitized sheep erythrocyte surface was 1000-fold higher than that required for C1 inhibition in the fluid phase [49]. To achieve C1-inh inhibition in NMO, one approach might be direct targeting of C1-inh to AQP4-expressing astrocytes in the brain, perhaps by C1-inh / AQP4 antibody fusions.

In conclusion, our in vitro and rat data in NMO models suggest that the complement inhibition activity of C1-inh in serum is far too low to produce significant complement inhibition and hence therapeutic benefit in NMO. Notwithstanding the similar activities of rat and human complement, and the similar C1-inh inhibition efficacy, studies done in experimental rat models should be extrapolated to humans with caution. Definitive evaluation of the potential efficacy of C1-inh in human NMO will require controlled clinical trials.
